# MiR-146b negatively regulates migration and delays progression of T-cell acute lymphoblastic leukemia

**DOI:** 10.1038/srep31894

**Published:** 2016-08-23

**Authors:** Nádia C. Correia, Rita Fragoso, Tânia Carvalho, Francisco J. Enguita, João T. Barata

**Affiliations:** 1 Instituto de Medicina Molecular, Faculdade de Medicina, Universidade de Lisboa, Lisboa, Portugal.

## Abstract

Previous results indicated that miR-146b-5p is downregulated by TAL1, a transcription factor critical for early hematopoiesis that is frequently overexpressed in T-cell acute lymphoblastic leukemia (T-ALL) where it has an oncogenic role. Here, we confirmed that miR-146b-5p expression is lower in TAL1-positive patient samples than in other T-ALL cases. Furthermore, leukemia T-cells display decreased levels of miR-146b-5p as compared to normal T-cells, thymocytes and other hematopoietic progenitors. MiR-146b-5p silencing enhances the *in vitro* migration and invasion of T-ALL cells, associated with increased levels of filamentous actin and chemokinesis. *In vivo*, miR-146b overexpression in a TAL1-positive cell line extends mouse survival in a xenotransplant model of human T-ALL. In contrast, knockdown of miR-146b-5p results in leukemia acceleration and decreased mouse overall survival, paralleled by faster tumor infiltration of the central nervous system. Our results suggest that miR-146b-5p is a functionally relevant microRNA gene in the context of T-ALL, whose negative regulation by TAL1 and possibly other oncogenes contributes to disease progression by modulating leukemia cell motility and disease aggressiveness.

Improved therapy regimens have led to cure rates close to 80% in children with acute lymphoblastic leukemia (ALL)^1^,^2^. Although risk-adjusted chemotherapy improved the outcome of ALL patients presenting with T-cell phenotype (T-ALL), these still have higher risk for induction failure, early relapse, and isolated CNS relapse^3^. Thus, a better understanding of the biology of the disease, namely through the molecular analysis of common genetic and epigenetic alterations, is necessary to the development of more efficacious and less toxic rationally-designed therapies.

MicroRNAs (miRNAs) are small, non-coding RNAs that primarily function as endogenous translational repressors of protein-coding genes^4^. Importantly, miRNAs are key regulators of cancer progression^5^,^6^,^7^, namely by modulating the expression of oncogenes and tumor suppressors and thereby inhibiting or promoting tumorigenesis. In ALL, microRNA expression signatures delineate leukemia subgroups^8^,^9^,^10^,^11^, and deregulated miRNA networks have been implicated in T-ALL^12^,^13^,^14^,^15^,^16^.

TAL1 is a transcription factor essential for the maintenance of hematopoietic stem cells and regulation of early hematopoiesis^17^,^18^,^19^,^20^. TAL1 expression is shut down upon T-cell lineage commitment^21^ and its aberrant expression in committed T-cell precursors is associated with leukemogenesis, with TAL1 overexpression occurring in more than 60% of T-ALL patients^22^,^23^,^24^. Although a considerable number of TAL1 downstream target genes have been identified to date^12^,^23^,^25^,^26^,^27^,^28^,^29^,^30^,^31^,^32^,^33^,^34^,^35^,^36^ validation and characterization of their functional involvement in TAL1-mediated leukemogenesis remain fragmentary.

Recently, it has been demonstrated that TAL1 regulates the expression of microRNA genes^37^,^38^. Prominent amongst these is miR-223, which is positively regulated by TAL1^37^,^38^ and highly expressed in T-ALL^13^. TAL1 transcriptionally activates miR-223 and thereby downregulates the tumor suppressor FBXW7^38^. However, whether other microRNAs are involved in TAL1-mediated T-cell oncogenesis has not been addressed. Several studies have implicated miR-146b-5p, which is inhibited by TAL1^37^, as having a tumor suppressor role in solid tumors^39^,^40^,^41^,^42^,^43^,^44^ and in human diffuse large B-cell^45^ and mouse PTEN-deficient T-cell^46^ lymphomas. In the present study, we show that miR-146b-5p is a functionally relevant TAL1 downstream microRNA target gene, whose downregulation contributes to T-ALL by impacting on leukemia cell motility *in vitro* and disease aggressiveness *in vivo*.

## Results

### MiR-146b is downregulated in T-ALL

**Figure 1:**
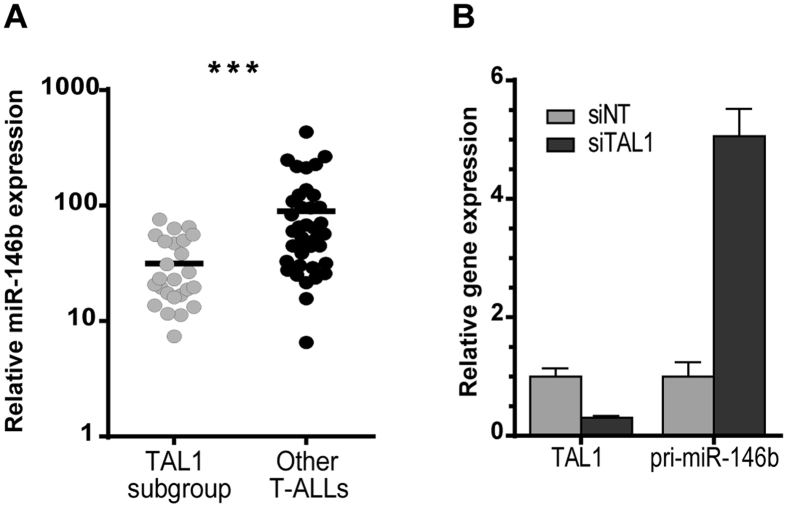
TAL1-positive T-ALL cells express low levels of miR-146b-5p and TAL1 silencing upregulates miR-146b-5p primary transcript. **(A)** miR-146b-5p expression in primary T-ALL cells. MiR-146b-5p levels were analyzed from publically available data^48^ in a cohort of 64 T-ALL patients comparing TAL1+ T-ALL cases (such as SIL-TAL, TCR-TAL and other TAL1+ cases – TAL1 subgroup) with T-ALL cases carrying other genetic abnormalities (TLX1, TLX3, HOXA and immature subgroups – Other T-ALLs). Statistical analysis was performed using Student’s t-test (***p < 0.001). **(B)** CEM cells were nucleofected with siRNAs against *TAL1* (siTAL1) or a non-targeting control (siNT) and the expression of *TAL1* (left) or pri-miR-146b (right) transcript was assessed by qRT-PCR. Values indicate the mean ± lower and upper limit of three technical replicates relatively to the siNT control.

**Figure 2:**
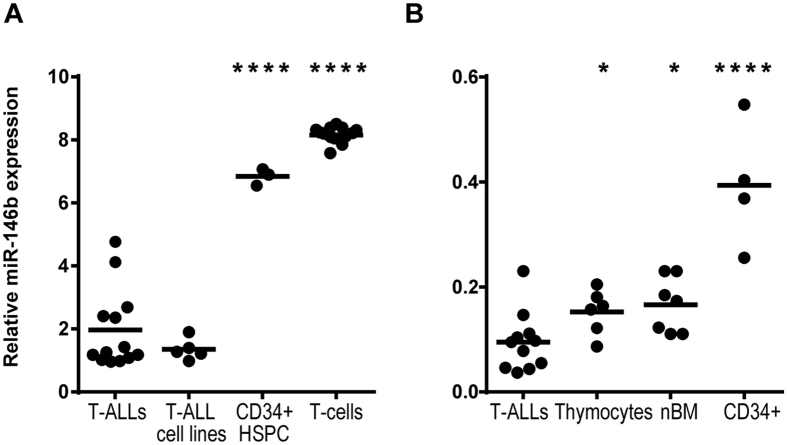
T-ALL cells express lower levels of miR-146b-5p than normal controls. **(A**,**B)** MiR-146b-5p expression in primary T-ALL samples was analyzed from publicly available data (GSE51908 and ref 10). **(A)** MiRNA expression in T-ALL patients and cell lines was compared to normal T-cells or CD34+ hematopoietic progenitor/stem cells (HSCP) cells from the peripheral blood of healthy donors. Data was collected from the GEO database (GSE51908). **(B)** MiRNA expression in T-ALL patients was compared to thymocytes, bone marrow (nBM) and CD34+ peripheral blood cells of pediatric samples^10^. Statistical analysis was performed using One-way ANOVA (*p < 0.05; ****p < 0.0001).

We previously showed that miR-146b-5p was downregulated by TAL1 in T-ALL cell lines and that TAL1-positive T-ALL patients tended to display reduced levels of miR-146b-5p as compared to other T-ALL cases^37^. Using a recently published miRNA expression dataset^47^,^48^ we found that pediatric T-ALL patient samples overexpressing TAL1 (TAL subgroup) had significantly lower levels of miR-146b-5p than samples carrying other genetic alterations (Fig. 1A). Furthermore, the knockdown of *TAL1* in a T-ALL cell line resulted in marked up-regulation of pri-miR-146b (Fig. 1B), indicating a strong negative impact of TAL1 on miR-146b expression. Notably, we also found that T-ALL primary cells and cell lines expressed significantly lower levels of miR-146b-5p than normal hematopoietic control cells, such as T-cells, thymocytes, bone marrow precursors and CD34+ hematopoietic progenitor/stem cells (Fig. 2). Overall, these observations led us to hypothesize that downregulation of miR-146b-5p is functionally relevant in the context of human T-ALL in general and especially in TAL1 overexpressing cases.

### MiR-146b inhibits motility, migration and invasion of T-ALL cells

Next, we sought to determine the functional consequences of miR-146b decreased expression in T-ALL. To this end, we stably knocked down miR-146b-5p in TAL1-negative (DND-41 and MOLT-4) T-ALL cell lines or overexpressed miR-146b-5p in TAL1-positive (JURKAT and CEM) cells (Figure S1). We found no significant differences in cell proliferation, as assessed by cell counts (Figure S2A,B) and thymidine incorporation (Figure S2C), either in normal culture conditions (10% FBS) or under serum starvation (0% FBS). This is in accordance with a previous study reporting that miR-146a/b enforced expression has no effects on the proliferation of KOPTK1, RPMI-8402, DND-41 or TALL-1 cells^16^. Moreover, no differences were found in T-ALL cell viability upon modulation of miR-146b expression (Figure S3). Given that miRNA-146b-5p was shown to be highly up-regulated during the later stages of thymocyte maturation^49^, we reasoned that modulation of its expression could have an effect on T-ALL cell differentiation. However, we monitored the cell lines for several weeks and none displayed changes in the stage of maturation in which they were blocked (Figure S4).

**Figure 3:**
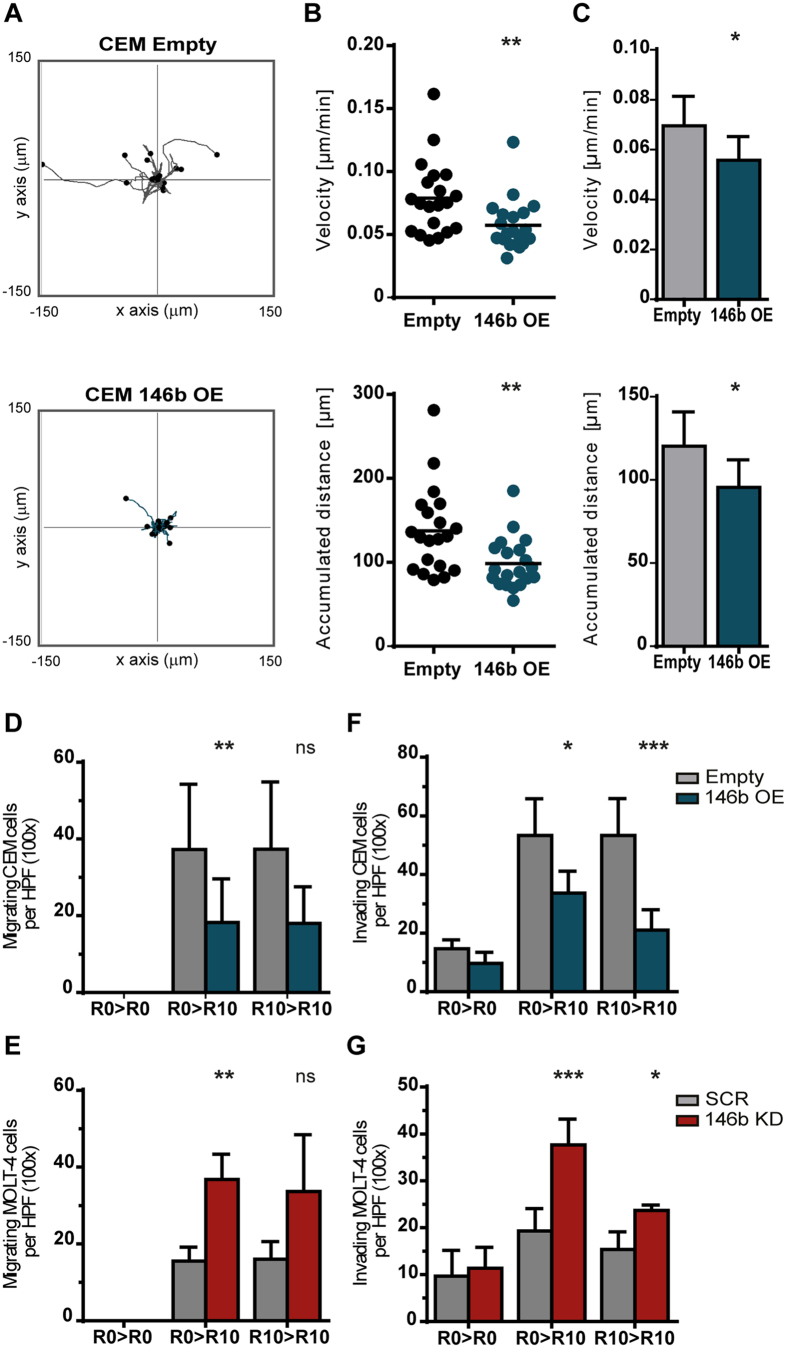
MiR-146b downregulates cell motility, migration and invasion of T-ALL cells. CEM cells ectopically expressing miR-146b (146b OE) or mock*-*transduced (Empty) were compared; MOLT-4 cells with downregulation of miR-146b-5p (146b KD) or scramble-transduced (SCR) were compared. Statistical significance was calculated using paired two-tailed Student’s t-test (*p < 0.05; **p < 0.01; ***p < 0.001). **(A)** Migration of individual CEM cells (n = 20) was recorded for 30 min by time-lapse video microscopy. The flower plot diagrams are representative of ten independent experiments. The starting point of each track is placed at the axis origin. **(B)** Migration velocity (top) and accumulated traveled distance (bottom) of leukemic cells depicted in (**A**). Lines indicate mean values. **(C)** The mean velocity (top) and accumulated distance (bottom) of 20 individual leukemic cells was assessed in ten independent time-lapse experiments. The bar graphs represent the mean ± SD. **(D–G)** Migration (**D,E**) and invasion (**F,G**) were assessed through transwell and matrigel coated transwell assays, respectively. Serum was used as chemoattractant. Cells were plated on the upper chamber of the transwell in culture medium in the absence (R0) or presence of 10% serum (R10), as indicated: medium present in the upper chamber > medium in the lower chamber (R10). The number of cells was determined by counting five non-overlapping high-power fields (HPF×100) per transwell. At least three independent experiments were performed (in triplicate). Graphs represent mean ± SD number of cells per HPF counted in at least three independent migration experiments.

**Figure 4:**
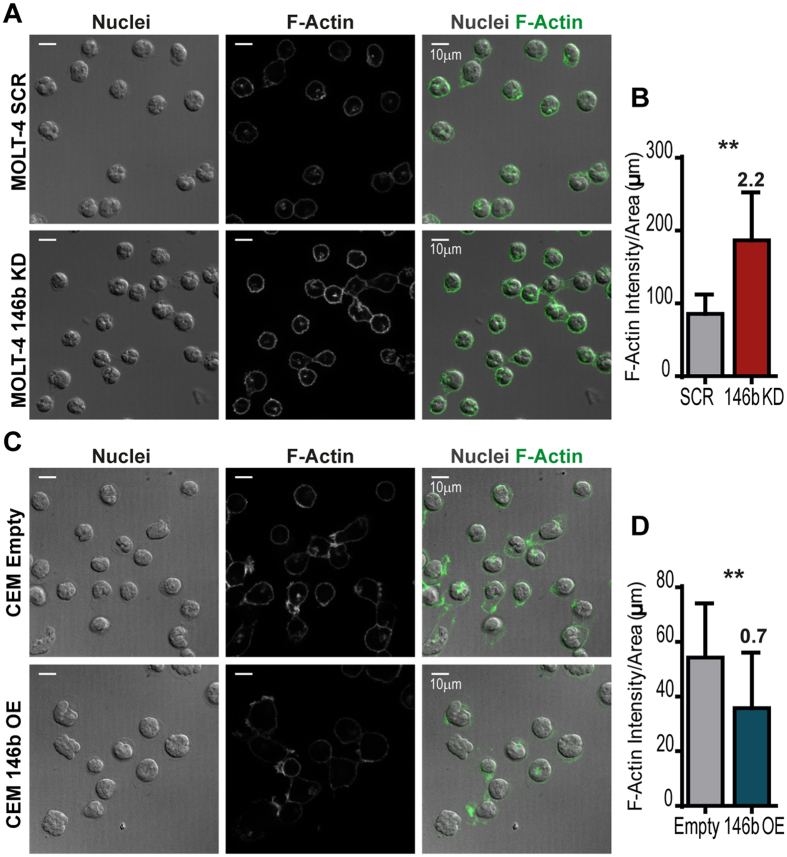
MiR-146b downmodulates actin polymerization in T-ALL cells. **(A**,**C)** Representative confocal images used for quantification of polymerized actin (F-Actin) through phalloidin-488 fluorescence intensity. The fluorescence of MOLT-4 cells (n = 30–50) with miR-146b-5p downregulation **(A**,**B)** or CEM cells with miR-146b overexpression **(C**,**D)** and respective controls was quantified in high-magnification (×63 objective) images. Scale bar: 10 μm. **(B**,**D)** The mean intensity of fluorescence per area was quantified in independent immunofluorescence staining preparations. The bar graphs depict the mean ± SD of 6 (MOLT-4) or 5 (CEM) independent experiments. The statistical significance of the differences observed was calculated using paired two-tailed Student’s t-test (**p < 0.01). The numeric values above each graph denote the average fold difference of F-actin per cell area in experimental conditions over those of control cells.

Altered expression of miR-146b has been linked to the migration properties of cancer cells in solid tumors^40^,^43^,^44^,^50^. Thus, we next investigated the functional impact of miR-146b on the motility and migration of T-ALL cells. Using time-lapse microscopy, we found that overexpression of miR-146b in TAL1-positive cells resulted in decreased cell motility (Fig. 3A–C), suggesting that the miRNA negatively affects random cell movement (chemokinesis). In addition, miR-146b reduced directional migration in response to serum, as assessed in transwell assays (Fig. 3D). On the contrary, downmodulation of miR-146b-5p in TAL1-negative T-ALL cells promoted migration under the same conditions (Figs 3E and S5). Notably, overexpression of miR-146b-5p in TAL1-positive T-ALL cells decreased their invasion ability (Figs 3F and S5), whereas silencing of miR-146b-5p in TAL1-negative cells had the opposite effect (Fig. 3G), as determined by cell migration through a matrix layer. In agreement with the impact of miR-146b on T-ALL cell movement, miR-146b-5p silencing led to increased actin polymerization (Fig. 4A,B). On the contrary, T-ALL cells overexpressing miR-146b exhibited lower levels of polymerized actin (Fig. 4C,D).

### MiR-146b delays leukemia progression *in vivo*

**Figure 5:**
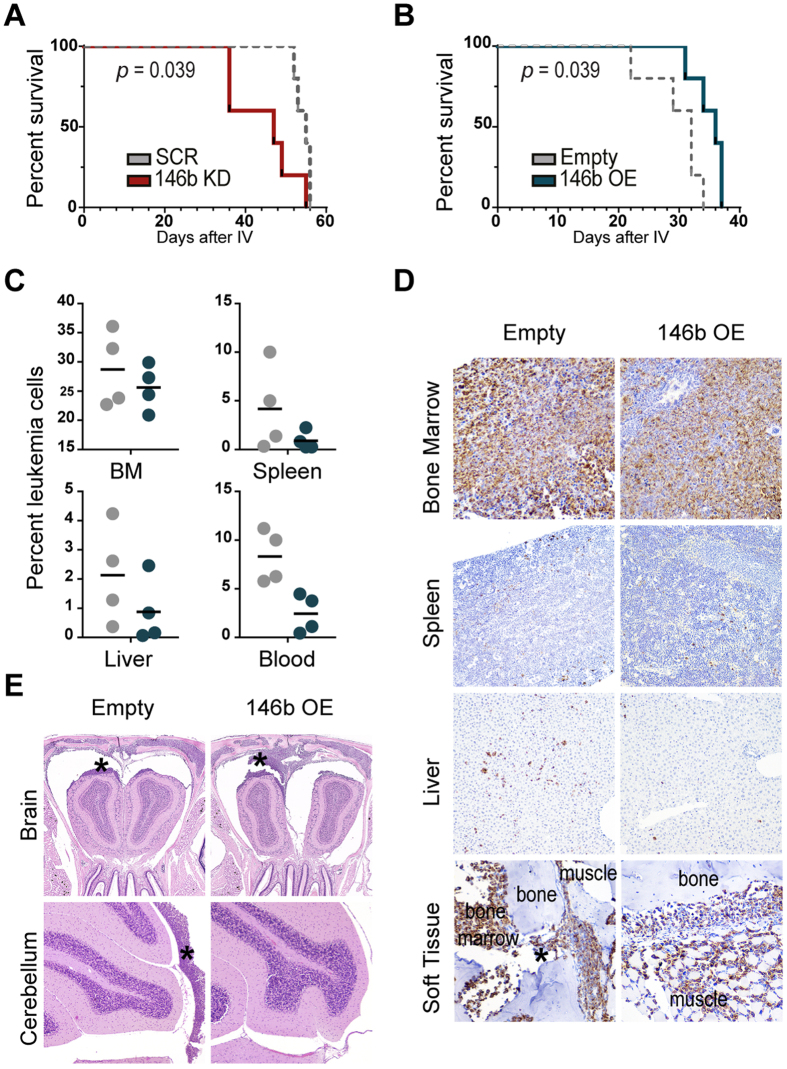
*In vivo* miR-146b-5p behaves as a tumor suppressor, with significant impact on T-ALL disease progression. NOD/SCID mice were xenotransplanted either with MOLT-4 cells with miR-146b-5p downregulation (146b KD; red) versus scramble transduced cells (SCR; grey) or with CEM cells ectopically expressing miR-146b (146b OE; blue) versus empty vector-transduced cells (Empty; grey). **(A**,**B)** Kaplan-Meyer survival curves, with a median survival of (**A**) 47 days for 146b KD (n = 5) versus 55 days for SCR control (n = 5), and (**B**) of 36 days for 146b OE (n = 5) versus 32 days Empty control (n = 5) (p < 0.05 in both cases, log-rank test). **(C–E)** Leukemia infiltration into different organs was assessed in CEM-transplanted mice (n = 4 Empty; n = 4 146b OE) at day 25 post-injection. **(C)** Erythrocyte-free cell suspensions of each organ were analyzed by flow cytometry to detect the presence of leukemic (RFP+) cells. The lines indicate the average percentage. BM – Bone marrow. **(D)** Representative micrographs of the bone marrow, spleen, liver and soft tissue. Immunohistochemistry for human vimentin was used to identify minimal infiltration by leukemia cells. Infiltration in the soft tissue originated from direct invasion from the bone marrow through the bone foramina of vertebra (*), skull, mandibular bones. 3,3′-Diaminobenzidine counterstained with hematoxylin; size units in μm; original magnification 200× (bone marrow and soft tissue), 100× (liver and spleen). **(E)** Representative H&E micrographs of CNS – brain (olfactory bulb) and cerebellum. In both experimental groups CNS infiltration corresponded to leptomeningeal infiltration, with the formation of multifocal to diffuse solid tumor masses (*) and with decreased severity in CEM 146b OE mice in comparison with controls. Size units in μm; original magnification 25× (brain) and 50× (cerebellum).

To investigate whether miR-146b exerts a tumor suppressor-like role *in vivo*, we used murine xenograft models of human T-ALL^51^. We transplanted MOLT-4 cells with stable silencing of miR-146b-5p or mock vector into immunocompromised mice. Silencing of miR-146b-5p in T-ALL cells significantly accelerated leukemia-associated death of transplanted mice (Fig. 5A). In contrast, miR-146b overexpression in CEM T-ALL cells delayed leukemia-associated death of transplanted mice (Fig. 5B), which presented decreased extent of infiltration of secondary organs (other than bone marrow) as compared to controls (Figs 5C–E and S6). For instance, a minimal infiltration pattern, with isolated cells, was observed for CEM cells with miR-146b overexpression, while empty vector-transduced cells showed a tendency towards the formation of larger foci of 5–10 cells (Fig. 5D). T-ALL cells overexpressing miR-146b also originated less severe leptomeningeal infiltration than control cells (Figs 5E and S6). Moreover, the frequency of leukemic cells in the blood reflected the pattern of leukemia spread, with a clearly lower percentage in the case of miR-146b-overexpressing cells (Fig. 5C). Altogether, these findings are consistent with the negative effect on motility we observed for miR-146b *in vitro* and with a tumor suppressor role for miR-146b-5p in T-ALL.

## Discussion

The identification and characterization of the full spectrum of TAL1-regulated genes, including microRNA genes, with functional impact on leukemia development has the potential to reveal novel molecular targets for therapeutic intervention. We previously showed that miR-146b-5p is negatively regulated by TAL1^37^. In the present study, we demonstrated that miR-146b-5p downmodulates motility, migration and invasion of T-ALL cells *in vitro* and leukemia dissemination and disease progression *in vivo*. Loss of miR-146a (which differs from miR-146b-5p by two nucleotides) in fetal liver hematopoietic progenitors overexpressing activated Notch does not appear to impact tumor onset in a mouse model of Notch-induced T-ALL^16^. Consequently, miR-146a/b have been discarded from a list of candidate tumor suppressor microRNAs in T-ALL. However, the inability of miR-146a to prevent leukemogenesis might be due to redundancy with miR-146b-5p, which is very abundant in hematopoietic progenitor cells. Alternatively, since miR-146a and miR-146b can also have specific, non-redundant targets and functions^46^, one cannot exclude that miR-146b may have a tumor suppressor role in human T-cells that is not embraced by miR-146a. Finally, it is plausible that miR-146a may be insufficient to prevent the activity of a very strong oncogene such as intracellular Notch. Nonetheless, in both our xenograft T-ALL models miR-146b modulation is sufficient to affect T-ALL development.

MiR-146b-5p is amongst the most expressed miRNAs in mature single-positive thymocytes^49^, being up-regulated during the double-positive to single-positive thymocyte transition^37^, consistent with a model whereby TAL1 aberrant expression contributes to leukemogenesis in developing thymocytes in part by downregulating miR-146b-5p. Nonetheless, our demonstration that T-ALL cells, irrespectively of their TAL1 status, express significantly lower levels of miR-146b-5p than healthy controls suggests that miR-146b-5p may be modulated by other factors in addition to TAL1. Identifying those factors may contribute to the growing understanding of the oncogenic pathways that underlie T-ALL and thus deserves further investigation.

Our demonstration that miR-146b-5p alters the motility, migration and invasion capacities of T-ALL cell lines *in vitro* is in agreement with previous findings in solid tumors^40^,^43^,^44^,^50^, including osteosarcoma (via AUF1 regulation)^39^, breast cancer^40^ (via NF-κB regulation)^41^, glioma (via MMP16^42^ and EGFR^43^ regulation), and pancreatic cancer (via MMP16 regulation)^44^. Evidently, our findings using leukemia cell lines warrant investigation in patient cells. Moreover, the question arises of which miR-146b-5p target(s) may be responsible for the effects we observed in T-ALL cells. Previously, we showed that miR-146b-5p validated targets are enriched in genes involved in biological processes such as inflammation (e.g., NF-kB and IL1/IL1R signaling pathways) and cancer^37^. Our current analyses, using GeneCodis^52^, extended to miR-146b-5p predicted target genes (n = 250, Table S1) and indicated that several migration-related processes are significantly enriched, including axon guidance, neural crest cell migration or regulation of actin cytoskeleton reorganization (Figure S7). In agreement, functional annotation analysis, using DAVID^53^, returned several gene ontology terms related to cell motility and migration that are significantly associated (p < 0.05) with miR-146b-5p predicted targets genes (Table S2). In particular, 50 out of 250 genes are implicated in biological processes such as cytoskeleton, cell migration, actin filament-based processes, and cell projections (Table S3). Thus, our bioinformatics analyses suggest that miR-146b-5p likely regulates cell motility and migration via multiple target genes.

Our *in vivo* findings suggest that miR-146b impacts the capacity of T-ALL cells to infiltrate hematopoietic and non-hematopoietic organs, thereby delaying leukemia progression and effectively acting as a tumor suppressor gene. MiR-146b has been implicated also in preventing proliferation of PTEN-deficient mouse CD4 thymocytes^46^ and human diffuse large B-cell lymphoma cells^45^. Moreover, several reports^41^,^46^,^54^,^55^ implicate miR-146b in decreasing NF-κB pathway activation in inflammation and cancer. In particular, miR-146b-5p has an anti-oncogenic function in the context of PTEN-deficient T-cell leukemia in mice that is mediated by attenuation of TCR signaling through direct repression of TRAF6. The consequence is inhibition of downstream NF-κB activation and c-Myc induction, associated with reduced proliferation^46^. However, our data suggest that miR-146b-5p does not have a similar role on proliferation of human T-ALL cells. In accordance, preliminary evaluation of NF-κB activation by assessment of the phosphorylation of IkBα and of RelA in our transduced T-ALL cell lines did not reveal any obvious differences modulated by miR-146b (data not shown). In addition, our studies using Ki-67 suggest that miR-146b-5p does not significantly affect human T-ALL cell proliferation *in vivo* (not shown). Although we cannot exclude the possibility that miR-146b-5p impacts leukemia cell survival *in vivo*, such an effect could still be the result of altered migration, which was shown to affect T-ALL cell viability in the bone marrow by regulating niche localization^56^,^57^,^58^.

From a therapeutic standpoint, it is noteworthy that intra-tumor injection of exosomes derived from miR-146b-expressing mesenchymal marrow stromal cells was shown to reduce glioma xenograft growth in a rat model of primary brain tumor^59^. Modulation of miR-146b appears to clearly affect CNS infiltration in our *in vivo* models of human T-ALL. In this context, it would be interesting to determine whether plasma levels of miR-146b in T-ALL patients correlate with CNS infiltration, and whether this could be transversal to other acute leukemias. Strategies involving carrier-based nanotechnology to enrich or antagonize miRNAs have been successfully experimented in murine lymphoma models^60^. When will these translate into clinical applications can only be speculated, but it is tempting to envisage future administration of miR-146b as a potential means to prevent or decrease CNS involvement, which is a major risk factor in T-ALL. In summary, we showed that miR-146b-5p downregulation promotes T-ALL by modulating leukemia cell motility, invasion, organ dissemination and consequent disease aggressiveness. These observations reveal a player in T-ALL biology and may help defining new therapeutic options in this malignancy.

## Methods

For detailed experimental procedures see online Supplemental Methods.

### Cell lines

Human T-ALL cell lines were maintained in RPMI medium (GIBCO) supplemented with 10% FBS at 37 °C with 5% CO_2_ and split every 2–3 days.

### Transduction of T-ALL cells for miR-146b overexpression or knockdown

VSVG-pseudotyped lentiviruses were produced by transient three-plasmid co-transfection into 293T cells. The vectors for miR-146b overexpression and respective control (pLemiR-146b-OE and pLemiR-empty) were kindly provided by the Sai Yendamuri lab. Both mature forms of miR-146b are expressed (miR-146b-5p and miR-146b-3p)^50^. The lentiviral vector for miR-146b-5p inhibition (146b_KD) is the pEZX-AM03 vector (Tebu-bio) and expresses the specific miRNA inhibitor against hsa-miR-145b-5p. The resulting cell lines expressing the vectors or the corresponding mock control were sorted for equivalent RFP expression.

### Assessment of proliferation

T-ALL cell lines were plated (5 × 10^5^ cells/mL) in triplicates in flat-bottom 96-well plates at 37 °C with 5% CO_2_ on day zero, either in RMPI-10 or RPMI-0 (no serum). Proliferation was measured at the indicated time points either by ^3^H-thymidine incorporation or by cell counts using a hemocytometer and trypan-blue for dead cell exclusion. Every other day, cells were counted and seeded as 5 × 10^5^ cells/mL.

### Assessment of cell viability

Cell viability was determined by flow cytometry analysis of Forward Scatter versus Side Scatter (FSCxSSC) distribution in an LSRFortessa cell analyzer (BD Biosciences).

### Cell migration and invasion assays

Cells (10^5^) were seeded in a 5μm pore transwell insert (Millipore) in RPMI medium either in the absence (R0) or presence of 10% serum (R10) and plated in a 24-well plate. Serum was added to the bottom chamber as a chemoattractant. For invasion assays, the transwell inserts were coated with a layer of Matrigel Growth Factor Reduced Matrix (BD biosciences).

### Immunofluorescence and time-lapse confocal microscopy

Distribution of F-actin was assessed using Alexa Fluor 488-phalloidin (Thermo Fisher). ICY software was used for 3D image reconstruction and fluorescence quantification of 30–50 cells per sample. For time-lapse video assessment of cell movements, phase-contract images were obtained every 60 s for 30 min at 37 °C with 5% CO_2_. Velocities and accumulated distance of 20 randomly selected leukemic cells were determined by manually tracking individual cells using manual tracking plugin on ImageJ (NIH) software.

### Human T-ALL *in vivo*

Age-matched (8 to 14 weeks) NOD/SCID mice were xenotransplanted with T-ALL cell lines and equally distributed by the experimental groups (n = 4 or 5 mice per group, as indicated). Experiments were performed once. Leukemia cells (10^7^) were injected in the tail vein. Experimental procedures were approved by the institutional Animal Ethics Committee from Instituto de Medicina Molecular and followed the recommendations for the care and use of laboratory animals from the European Commission and Portuguese authorities.

### Statistical analysis

All analyses were performed using GraphPad Prism version 6.01 (GraphPad Software). Significance was set for p < 0.05 (*p < 0.05; **p < 0.01; ***p < 0.001: **** p < 0.001).

## Additional Information

**How to cite this article**: Correia, N. C. *et al*. MiR-146b negatively regulates migration and delays progression of T-cell acute lymphoblastic leukemia. *Sci. Rep.* **6**, 31894; doi: 10.1038/srep31894 (2016).

## Supplementary Material

Supplementary Information
