# Supplementary material for: MiR-146b negatively regulates migration and delays progression of T-cell acute lymphoblastic leukemia
Source: Sci Rep. 2016 Aug 23;6:31894. doi: 10.1038/srep31894 (PMC4994040; doi:10.1038/srep31894)
Supplement: Supplementary Information [file srep31894-s1.pdf]

MiR-146b negatively regulates migration and delays progression of T-cell acute lymphoblastic leukemia

Nádia C. Correia, Rita Fragoso, Tânia Carvalho, Francisco J. Enguita, João T. Barata

Instituto de Medicina Molecular, Faculdade de Medicina, Universidade de Lisboa

SUPPLEMENTARY DATA

Supplementary Methods

Supplementary Figures S1-S6

Supplementary Tables S1-S5

Supplementary references

Supplementary Methods

MicroRNA nomenclature and annotation. Official nomenclature and sequence annotation for the relevant microRNAs is based on the miRBase version 21 (2014)[1].

RNA extraction, RT-PCR and quantitative-PCR. RNA was extracted using TRIZOL (Life Technologies Corporation) according to the manufacturer's instructions. The RT-PCR reaction was performed with up to 1µg of total RNA, using thereverse transcriptase SuperScript II (Invitrogen) and random hexamers, according to the manufacturer's instructions. Real time PCR was performed with Power SYBR Green (Applied Biosystems) and the primers used are indicated in **Table S4**. Expression of each gene was normalized to the expression level of the ribosomal RNA *18S* using the dCt method. To evaluate the fold difference of the gene of interest between samples, the ddCt method was used. For the detection of mature human miR-146b-5p expression, amounts ranging from 100-1000ng of total RNA were reverse transcribed using miRCURY LNA™ Universal RT kit (Exiqon). Real time PCR was performed with commercially available LNA-based primers (Exiqon) and SYBR Green (Exiqon). The relative expression of each microRNAs was normalized to *SNORD38B* expression using the dCt method. All PCR amplifications were performed in a ViiA7 Real-Time PCR System (Life Technologies) with a melting curve protocol to verify the primers' specificity.

Immunoblot. Cell lysates were prepared as described[2]. The following antibodies were used: Tubulin (Sigma, Clone DM 1A) and TAL1 (Milipore, clone BTL73). Densitometry analysis was performed using Adobe Photoshop CS5 Extended software.

Production of VSVG-pseudotyped lentiviruses. Vesicular-Stomatitis-Virus-pseudotyped third-generation lentiviruses were produced by transient three-plasmid co-transfection into 293T cells. Briefly, 4×10^6 293T cells were seeded in a 6 cm dish and transfected when 70-80% of confluence was reached. The plasmids were transfected using Lipofectamine

2000 (Life technologies), according to the manufacturer's protocol. A total of 3µg of the envelope plasmid pMD2.VSVG, 6µg of packaging plasmid psPAX2 and 9µg of the vector plasmid of interest were used for transfection. The culture medium was replaced after 14 to 16h with 6ml of fresh medium containing 20mM HEPES buffer (pH=7.9). The conditioned medium (Lentiviral Supernatants) was collected after another 24h and 48h. The supernatant was filtered through 0.45mm pore-size cellulose acetate filters, flash frozen in liquid nitrogen and stored at -80°C until use.

Transduction of T-ALL cells for miR-146b over-expression or knockdown. For construction of the miR-146b over-expression vector (pLemiR-146), the pLemiR lentiviral vector (Open Biosystems), was modified by inserting the pre-microRNA-146b sequence in the 3′ untranslated region of the gene encoding the TurboRFP red fluorescent protein and driven by the constitutively active cytomegalovirus (CMV) promoter[3]. Both mature forms of miR-146b are expressed from the pre-microRNA-146b (miR-146b-5p and miR146b-3p)[3]. The control vector condition refers to the pLemiR vector without an insert. The TAL1-expressing JURKAT and CCRF-CEM cells were transduced with these vectors. Briefly, 2.5 x 10^5 cells were incubated in 500µl RPMI-10 in a 24-well-plate with 500µl of lentiviral supernatant and 8ng/µl of polybrene (Sigma). Cells were spun down for 120min at 32°C at 2300 rpm. The resulting transduced cell lines were sorted for an equivalent RFP expression. The lentiviral vector for miR-146b-5p inhibition (named as 146b_KD) is the pEZX-AM03 vector (Tebu-bio), expressing the specific miRNA inhibitor against hsa-miR-146b-5p (the sequence is proprietary) under the control of the H1 promoter. An independent CMV promoter drives the expression of the reporter gene (mCherry). The control vector (named SCR) expresses a scramble miRNA inhibitor control sequence. The TAL1-negative DND4.1 and MOLT-4 cells were transduced with these vectors using the protocol described above.

Assessment of proliferation. T-ALL cell lines were plated (5 x 10^5 cells/mL) in triplicates in flat-bottom 96-well plates at 37°C with 5% CO2 on day zero. Proliferation was measured

either by thymidine incorporation or by cell counts. Regarding the first technique, it determines proliferation by analysis of DNA synthesis, which is assessed by ^{3}H-thymidine incorporation using a β-scintillation counter. Briefly, cells were incubated with ^{3}H-thymidine (1µCi/well) for 8h prior to harvesting at indicated time-points. Proliferation was also assessed by counting the cells in a hemocytometer and using trypan-blue for dead cells exclusion. Cell counts were performed at the indicated time points. In these experiments cells were plated in RMPI-10 and also RPMI-0 (no serum). Every other day, cells were counted and seeded as 5×10^5 cells/mL, so that medium loss would not compromise their proliferation rate.

Flow Cytometry. Standard procedures were used to stain the cells with fluorochrome-conjugated antibodies or to verify reporter protein expression. The antibodies used in this study were CD1a-APC, CD3-PerCP-Cy5.5, CD4-PE-Cy7, CD8-FITC, CD45-APC (eBioscience). Samples were acquired in LSRFortessa cell analyzer (BD Biosciences), unless stated otherwise. Data analyses were performed using the FlowJo software.

Cell migration and invasion assays. Cell migration and invasion studies were done in 24-well transwell cell culture chambers with the upper chamber containing filters of 5µm pore size (Millicell-24 Cell Culture Insert Plate, polycarbonate, Millipore). Cells (10^5) were re-suspended in 100µl of RPMI either in the presence of 10% (R10) or in the absence of serum (R0) and added to the upper wells. In the bottom chamber, 800µl of R10 or R0 was added. In each migration or invasion experiment the following conditions were always used: (1) R0 in the upper and lower chambers (R0>R0) - negative control, indicative of the 'constitutive' migration/invasion of the cells. (2) R0 in the upper chamber and R10 in the lower chamber (R0>R10) - experimental condition. FBS is used as a chemoattractant to evaluate directional migration. (3) R10 in the upper and lower chambers (R10>R10) - experimental condition. The chemoattractant is present in both chambers to evaluate random migration. Each condition was performed in triplicates per assay. Transwells were incubated at 37°C for the appropriated time (DND-41 cells two hours; CCR-CEM and

MOLT-4 cells three hours). To perform the invasion assays, the upper chamber was coated with a layer of 200µl of 1mg/ml of Matrigel Growth Factor Reduced Matrix (BD biosciences) that was let to solidify overnight at 37°C prior to the assays. The experimental conditions tested were the same described above for the migration assays, except for the incubation time that was longer, 7 to 8 hours. The number of migrating/invading cells was determined by counting the cells in the bottom of the lower chamber in a white field microscope (100x magnification) in five non-overlapping observation fields. The average number of cells per high-power field (HPF) of the five observation areas was determined in three technical replicates per condition. To calculate the migration index, the average number of cells per HPF was compared to those of the mock transduced cells and the fold difference of migrating cells calculated for each independent migration experiment. Unless stated otherwise, the average migration index of several independent experiments was used to plot the results and to calculate the significance of the differences observed between the miR-146b-5p modulation and the mock control cells.

F-Actin fluorescent staining and time-lapse confocal microscopy. Distribution of the F-actin was assessed using Alexa Fluor 488-phalloidin (Thermo Fisher). First, cells were let to adhere to PDL-coated coverslips for 10 min. Then, cells were fixed in 2% PFA for 10 min and permeabilized with 0.1% Triton X-100 in PBS for 5 min. The coverslips were stained for 30 min at room temperature with Alexa Fluor 488–phalloidin in PBS. Images were obtained using a 63× oil immersion objective in the SDC spinning disk microscope and z stacks were generated from optical sections taken at 0.27-µm intervals. Several images per coverslip were taken in order to sample sufficient cells. ICY software[4] was used for 3D image reconstruction and fluorescence quantification of 30-50 cells per sample. The Maximum intensity projection of the green channel was calculated for every 3D image. Regions of interest comprehending 30-50 cells were defined and the mean intensity of the green channel per area was calculated for each independent experiment. For time-lapse video assessment, $3x10^3$ cells were plated in serum-free medium in fibronectin (Sigma) coated µ-slides angiogenesis (IBIDI) and let to adhere for 30 min at 37ºC. Right before

image acquisition RPMI with 10% FBS was added to the cells. Phase-contract images were obtained every 60s for 30 min at 37°C with 5% CO2 in a SDC spinning disk microscope using a 20x objective. Velocities and accumulated distance of 20 randomly selected leukemic cells were determined by manually tracking individual cells using manual tracking plugin on ImageJ (NIH) software.

miRNA target prediction and gene set enrichment analysis. Prediction of microRNA putative targets was performed by TargetScanS release 4.2 [5] and DIANA-microT algorithm V3.0 [6]. MicroRNAs experimentally validated human targets were obtained from mirTARbase 3.5, miRrecords and TarBase 6.0. Target genes without matching any Entrez gene identifier in NCBI were discarded. For gene set enrichment analysis we performed Singular Enrichment Analysis of GO Biological Process using GeneCodis (http://genecodis.cnb.csic.es/). P-values were determined by a hypergeometric test, followed by a false discovery rate correction to account for multiple hypotheses. For biological function and pathway analysis, a Functional Annotation Chart analysis was performed using David web-based bioinformatic (https://david.ncifcrf.gov/). p-values were determined by a modified Fisher Exact Test for gene-enrichment analysis (p-Value is equal or smaller than 0.05 to be considered strongly enriched in the annotation categories). The significantly enriched GO terms related to cell motility and migration (cytoskeleton, cell migration, actin filament-based processes and cell projections) were manually curated to create a list of genes (n=50) predicted to be targeted by miR-146b-5p and potentially involved in the observed phenotype.

Xenotransplant mouse models Age-matched males and females (8 to 14 weeks) NOD/SCID mice were used in these experiments and equally distributed by the experimental groups. For the survival curve, four experimental groups were established: MOLT-4 SCR and MOLT-4 146b_KD, and CCRF-CEM empty and CCRF-CEM 146b_OE (each group n=5). Leukemia cells (10^7) were injected intravenously in the tail. For the analysis of mouse overall survival humane endpoints were established. In particular, mice were

euthanized when presenting a 20% weight loss or signs of lethargy. These endpoints were used to build the survival curve. The Kaplan-Meier estimator was used to determine the median rate of survival. The p-value was determined using the Log-rank (Mantel-Cox) test. To further assess T-ALL development, peripheral blood samples were collected weekly from the recipient mice (via facial vein puncture) starting two weeks after transplantation. The presence of human T-ALL cells was determined by flow cytometry analysis of RFP positive cells. Count beads (BD Biosciences) were used to determine the absolute number of RPF+ cell per volume of blood. At the time of the humane endpoints mice were sacrificed with anesthesia overdose (Isoflurane) and selected organs were collected for histopathology analysis. For leukemia cell infiltration characterization: CCRF-CEM 146b_OE and respective control cells were injected (10^7) in the tail vein of NOD/SCID mice (n=4). Animals from both groups were sacrificed at a pre-defined early time point of leukemia development (day 25). Selected organs were collected for histopathology and cytometry analysis. For FACS analysis, the liver, spleen, long bones and blood (cheek puncture) were collected. Single-cell suspensions were obtained by gridding the organs or, in the case of the BM, by flushing-off the tibias and femurs with PBS-2%FBS. When present, erythrocytes were lysed using the RBCL buffer (eBiosciences), following the manufacturer's instructions. The erythrocyte-free cell suspension was then acquired on a FACSAria III (BD Biosciences) to detect the presence of RFP+ cells.

Histopathology. Selected organs [liver, spleen, lung, kidney, lymph nodes, thymus, femur and tibia, and central nervous system (brain, cerebellum and spinal cord)] were harvested, fixed in 10% neutral-buffered formalin, embedded in paraffin and 3µm sections were stained with hematoxylin and eosin (HE). Bones were further decalcified in Calci-Clear™ (Fisher Scientific) prior to embedding. Immunohistochemical staining for Vimentin (mouse anti-human, clone V9, Dako, cat. no. M0725) and Ki-67 (mouse anti-human, Clone MIB-1, Dako, cat. no. M7240) was performed using standard protocols. Antigen heat-retrieval was performed in DAKO PT link with low pH solution (pH 6), and incubation with ENVISION kit (Peroxidase/DAB detection system, DAKO, Santa Barbara, CA) was followed by Harris's

hematoxylin counterstaining (Bio Otica, Milan, IT). Negative control included the absence of primary antibodies. No cross-reactivity with mouse tissue/cells was observed for any of the antibodies, and vimentin immunostaining was used to assess minimal/single-cell infiltration in secondary organs. Tissue sections were examined by a pathologist blinded to experimental groups, in a Leica DM2500 microscope coupled to a Leica MC170 HD microscope camera. All organs were assessed for leukemia cell infiltration, and the extent of infiltration was scored using a semi-quantitative 5-point severity scale, according to criteria shown in **Table S5**. For central nervous system (CNS), the infiltration pattern corresponded to invasion of the leptomeninges, without parenchymal infiltration. Coronal/axial sections of olfactory bulb, brain at hippocampus, cerebellum and cervical, thoracic, lumbar and sacral spinal cord were examined and animals were scored positive for CNS invasion when tumor cells were seen in at least one of these sites. The soft tissue invasion evaluation corresponds to masticatory and lumbar muscles, and adjacent connective tissue.

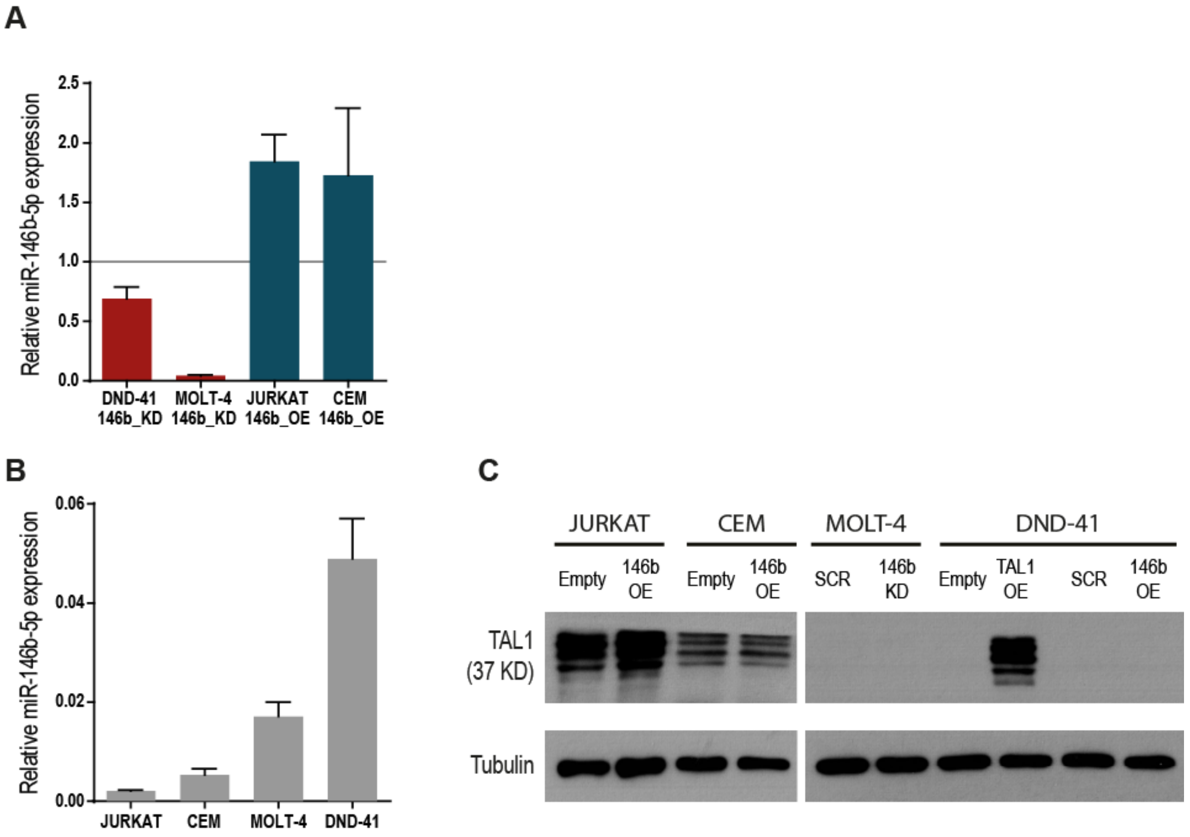

Figure S1. miR-146b-5p expression in transduced T-ALL cell lines. (A,B) miR-146b-5p levels were assessed by qRT-PCR and normalized to *SNORD38*. Bars indicate the mean ± SD of three independent RNA extractions. **(A)** The TAL1-negative cell lines (DND-41 and MOLT-4) transduced with a lentiviral vector for miRNA inhibition (146b_KD) express lower levels of miR-146b-5p. The TAL1-positive T-ALL cell lines (JURKAT and CCRF-CEM) transduced with pLemiR-146b vector (146b_OE) over-express miR-146b-5p. The graph depicts the fold difference of miRNA expression to the respective mock transduction. **(B)** Basal miR-146b-5p expression levels in the WT cell lines assessed by qRT-PCR and normalized to *SNORD38.* **(C)** Immunoblot analysis of the transduced T-ALL cell lines to confirm TAL1 expression. Tubulin was used as loading control. In the right panel, DND-41 cells over-expressing TAL1 were used as positive control of the TAL1 protein expression.

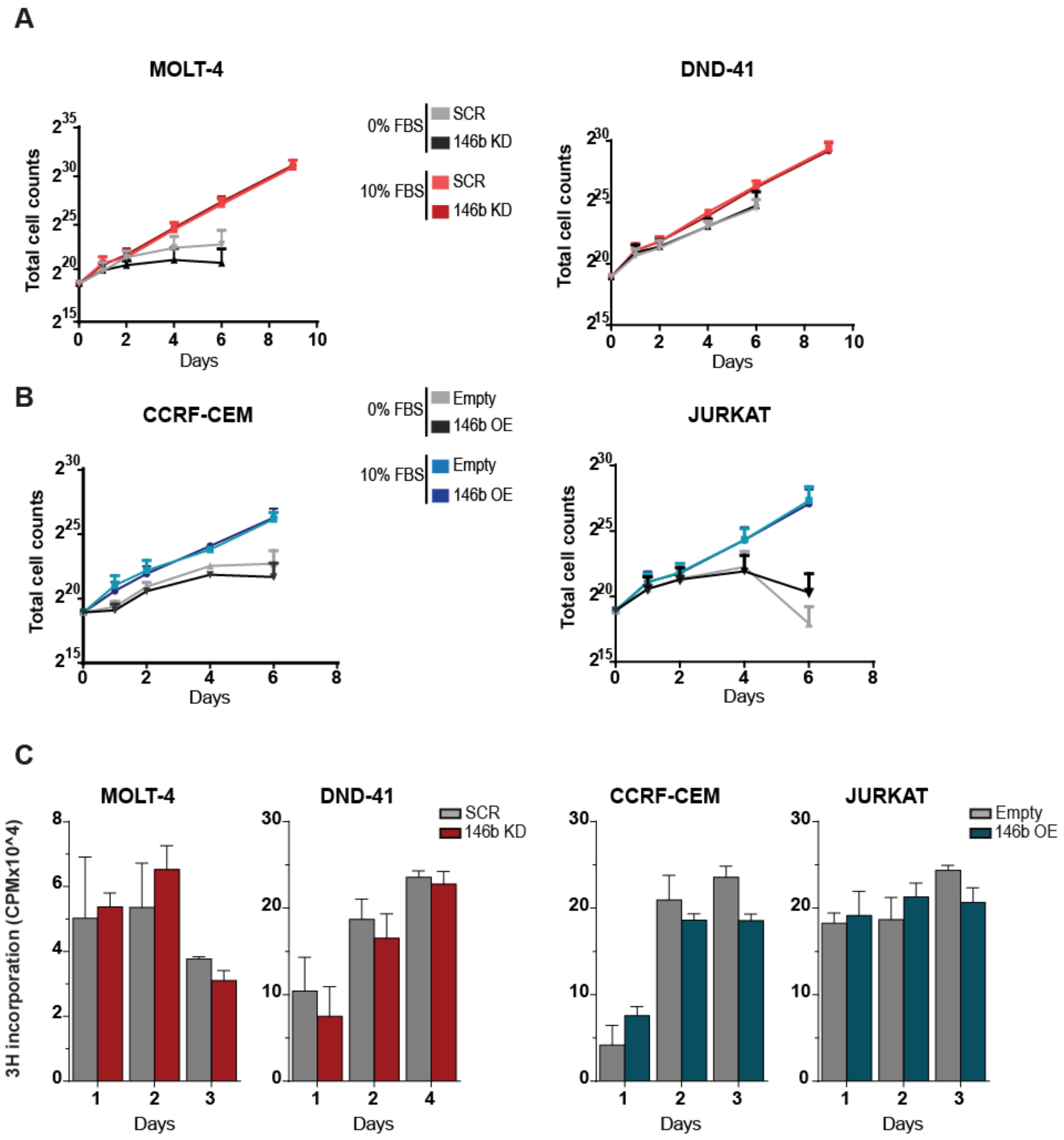

Figure S2. miR-146b modulation has no effect on proliferation of T-ALL cells. Proliferation of TAL1-negative T-ALL cell lines MOLT-4 and DND-41 with miR-146b-5p down-regulation (146b KD) **(A)** and of TAL1-positive cell lines CCRF-CEM and JURKAT with miR-146b over-expression (146b OE) **(B)** was determined by cell counts at the indicated time points either in regular medium (10% FBS) or under serum deprivation (0% FBS). Data represent the mean (± SD) of three independent experiments. **(C)** Proliferation of the same T-ALL cell lines was also assessed by ^{3}H-thymidine incorporation. The bar graphs represent the mean (± SEM) of three technical replicates.

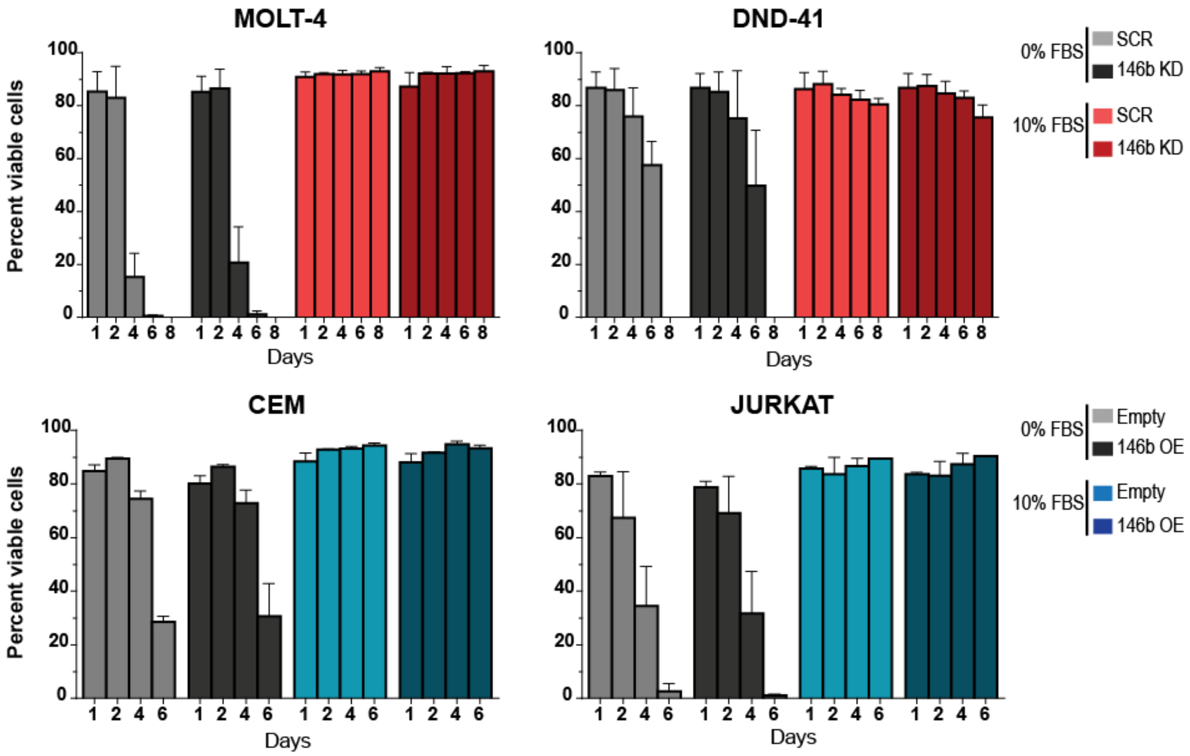

Figure S3. miR-146b modulation has no effect on the viability of T-ALL cells. Viability of MOLT-4 and DND-41 cell lines expressing lower levels of miR-146b-5p (146b KD) and of CCRF-CEM and JURKAT cell lines ectopically expressing miR-146b (146b OE) was assessed by flow cytometry analysis of Forward Scatter versus Side Scatter (FSCxSSC) distribution. The bars graphs represent the mean (± SD) of three independent experiments.

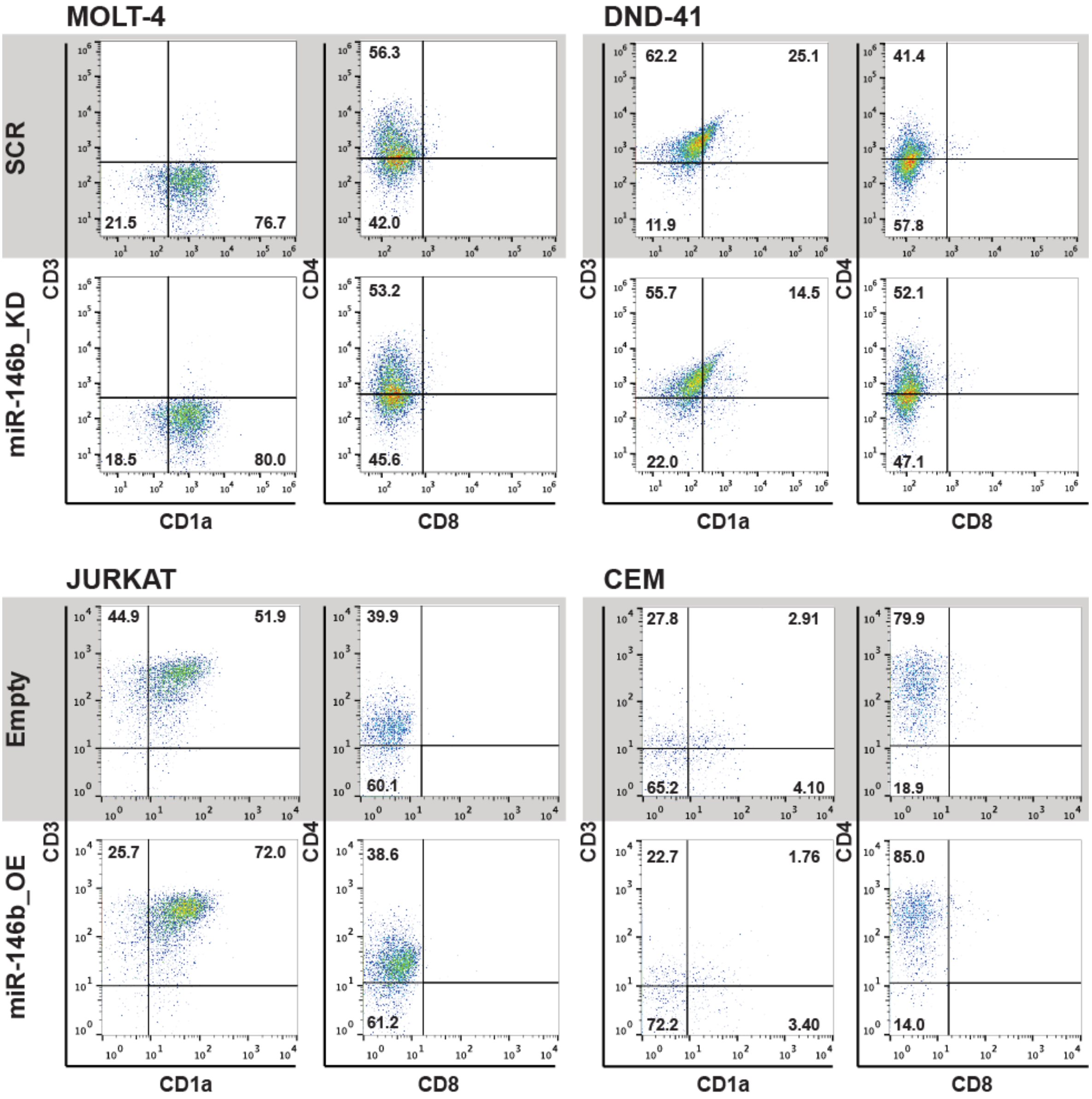

Figure S4. Immunophenotypic characterization of transduced T-ALL cell lines. Expression of the surface markers CD1a, CD3, CD4 and CD8 was accessed by flow cytometry (FACS) in the transduced T-ALL cell lines in order to determine their immunological phenotype. The cell lines had been stably transduced for more than two months at the time of the analysis. Shown are representative FACS plots of cell lines (MOLT-4 and DND-41) with down-regulation of miR-146b-5p (146b_KD) and respective SCR control and also the cell lines (JURKAT and CCRF-CEM) with miR-146b over-expression (146b_OE) and correspondent Empty control.

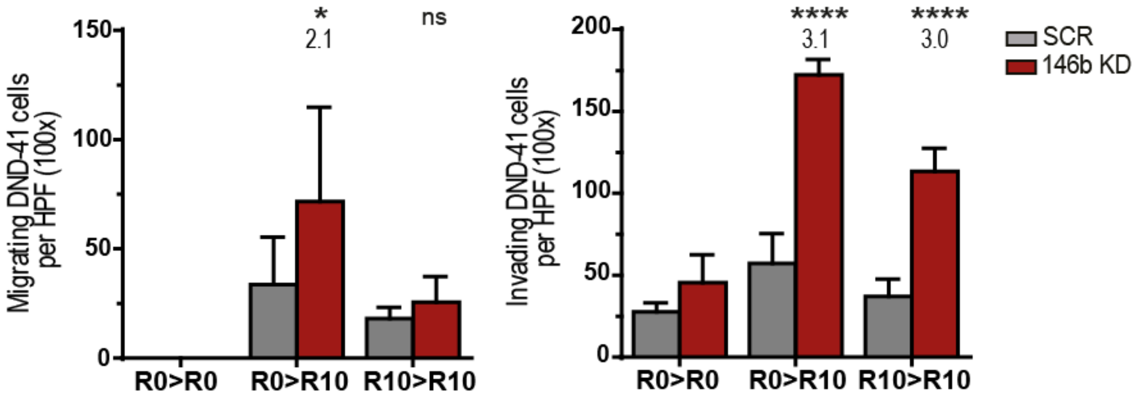

Figure S5. Down-regulation of miR-146b-5p enhances migration and invasion of DND-41 cells. T-ALL DND-41 cells with down-regulation of miR-146b-5p (146b_KD) were compared to mock transduced controls (SCR). Migration (left) and invasion (right) were assessed through transwell and matrigel coated transwell assays, respectively. Serum was used as chemoattractant. Cells were plated on the upper chamber of the transwell in culture medium either in the absence (R0) or in the presence of 10% serum (R10), as indicated: medium present in the upper chamber > medium in the lower chamber. After 2h (migration) or 7h (invasion), the number of migrating or invading cells per high-power field (HPF 100x) was determined by the average number of cells counted in five non-overlapping microscope fields of the same transwell. Cells were counted in technical triplicates. All experiments were performed at least three independent times. Symbols depicted represent the p-values calculated using two-tailed Student's t-test (*$p<0.05$; ****$p<0.0001$). (Left) The graph depicts the mean (± SD) number of migrating cells per HPF counted in at least three independent migration experiments. The numeric represent the average fold difference of migrating cells in independent experiments over the mock transduced cells. (Right) The graphs represent the mean (± SD) number of invading cells per HPF counted in triplicates of one representative experiment of invasion.

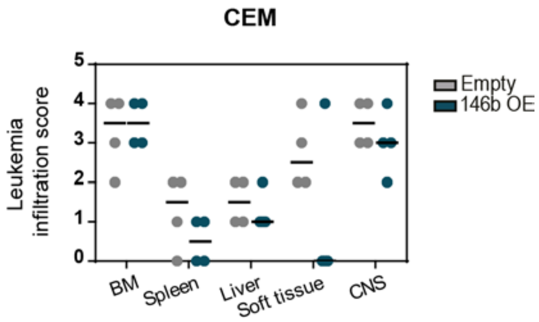

Figure S6. Leukemia cell infiltration determined by immunohistology. CEM leukemia cell infiltration score of bone marrow (BM), central nervous system (CNS; brain, cerebellum and spinal cord), liver, soft tissue and spleen was evaluated at day 25 (n=4 each group), as detailed in the Supplemental Materials and methods and in Table S5. Lines represent the median score. Data in this figure relates to Figure 5C-E.

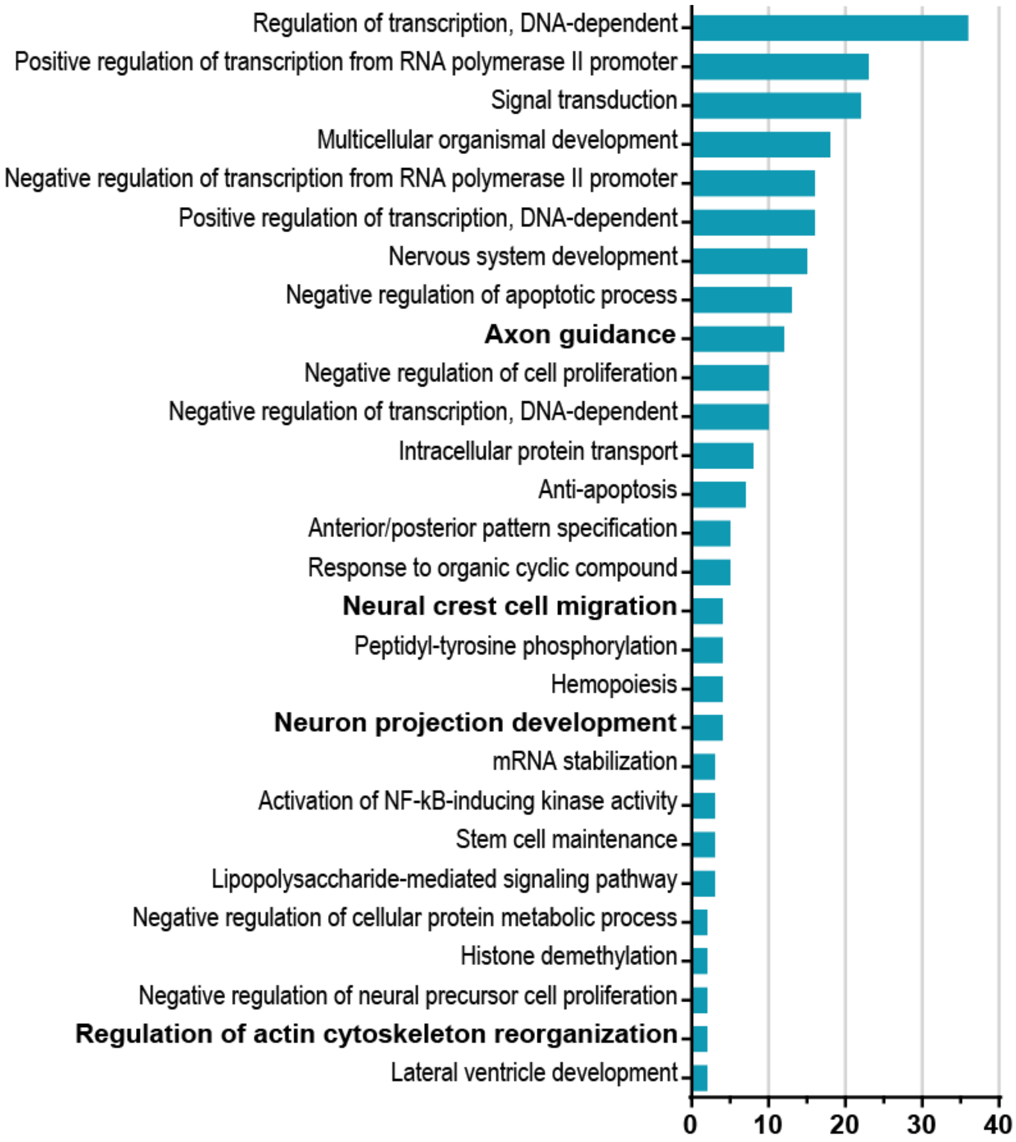

Figure S7. Graphical representation of gene ontology analysis of miR-146b-5p predicted target genes. Gene set enrichment analysis was performed using GeneCodis (http://genecodis.cnb.csic.es/). Here we represent the results for Singular Enrichment Analysis of GO Biological Process. In the y axis are plotted the biological processes that were found to be significantly enriched among the 250 predicted targets. In the x axis is plotted the number of genes among the predicted targets that are involved in the specific biological process. P-values were determined by a hypergeometric test, followed by a false discovery rate correction to account for multiple hypotheses. Only processes with p-values lower than 0.05 were considered to build the graph.

Table S1. List of human miR-146b-5p predicted targets.

Data obtained from TargetScanS release 4.2 and DIANA-microT V3.0 web-based bioinformatic tools. Number of predicted target genes = 250.

Tool	Predicted Target Genes miR-146b-5p
TargetScanS & DIANA-microT	ABL2, ACKR2, ADARB1, AKAP9, ALX4, AP3M2, APOL6, APPL1, ARL17, ARL8A, ARMC8, ATG12, ATG7, BAG1, BCL11A, BCORL1, BEND4, BIVM, BNC1, BTG2, C10orf76, C17orf75, C17orf78, C4orf3, C5orf46, C6orf203, CAMSAP1, CAPN14, Card10, CASK, CASZ1, CCDC6, CCNJ, CD80, CDC42BPA, CDKN1A, CDON, CDS1, CELF3, CHML, CNTFR, CSF1R, CUX1, DCAF12, DCDC5, DCTN5, DDHD1, DGKG, DLGAP2, DNAL1, DNPEP, DTNA, DYNLL2, EARS2, EDNRB, EHF, EIF4G2, EIF5A2, ELAVL1, ERBB4, ESYT2, FAF2, FAM120AOS, FAM210A, FAM26E, FAM43A, FAM65B, FAXDC2, FBXO28, FBXW2, FLOT2, FRYL, FZD1, GABPA, GALNT10, GAS7, GDNF, GGA2, GJA7, GLYATL3, GNL1, GOSR1, GRID1, GRIN2A, GRSF1, HIC2, HIF3A, HNRNPD, HNRNPUL2, IER5L, IGSF1, IRAK1, ITGAV, JAZF1, KBTBD3, KCMF1, KCNIP3, KCTD15, KDM2B, KDM6B, KIAA0284, KIAA1274, KIF24, KIT, KLF7, KPNA6, LCOR, LFNG, LIN28A, LPGAT1, LRP2, LRRC15, LRRC27, LRRTM2, LRTOMT, LYRM2, MAPT, MED1, MED20, METTL21A, MICU1, MLL2, MLXIP, MMP16, MOB1B, MPPE1, MTAP, MTDH, MYBL1, MYO5A, MYO6, MYT1, NAIF1, NF2, NFASC, NFAT5, NFIX, NFKB1, NOS1, NOTCH2, NOVA1, NPAS4, NPR3, NRAS, NRIP3, NRP2, NSD1, NUCKS1, NUMB, ONECUT2, PAQR9, PBX2, PCDH1, PHF20L1, PHOX2B, PLEKHH2, PM20D2, POFUT2, PPM1A, PPP1R11, PRC1, PRKCE, PSMD3, PTGFRN, QKI, RABGAP1, RCSD1, RFTN2, RIMS2, RNASEL, RNF4, ROBO1, RRM2B, RUNX1T1, SBSPON, SCN3B, Scube2, SDK1, SEC23IP, SEMA3G, SEPT3, SFRS6, SH3GL2, SH3TC2, SIAH2, SIN3A, SLC10A3, SLC2A3, SLC38A1, SLC39A1, SLCO3A1, SLITRK3, SLITRK4, SMAD4, SMARCA5, SMYD5, SNX21, SORT1, SOX5, SP8, SRP14, SRRM4, SRSF12, SSTR1, ST8SIA4, STC1, STRA13, STRBP, STRN, STX3, SYT1, TAF9B, TANC2, TDRKH, THAP3, TLCD2, TLN2, TMEM120B, TMEM136, TMEM194A, TMEM257, TMEM33, TRAF6, TRDMT1, TTL, TTPAL, UHRF1, USP3, USP47, VASN, VAT1, VBP1, WASF2, WASF3, WWC2, XIAP, YLPM1, ZBTB2, ZC3H12B, ZC3H6, ZDHHC7, ZFYVE1, ZNF148, ZNF367, ZNF512B, ZNF532, ZNF605, ZNF652, ZNF678, ZNRF3

Table S2. Functional Annotation Chart (DAVID) analysis of predicted miR-146b-5p target genes (Table S1).

Gene set enrichment analysis was performed using David web-based bioinformatics (https://david.ncifcrf.gov/). p-values were determined by a modified Fisher Exact Test for gene-enrichment analysis (p-value is equal or smaller than 0.05 to be considered strongly enriched in the annotation categories). The GO terms related to cell motility and migration that are significantly enriched were manually curated to create this and Table S3. The third column depicts the number of genes involved in the term; the fourth column depicts the relative percentage of the genes involved (total n=250).

Category	Term	Nr of genes	%	P -Value
GOTERM_CC_FAT	GO:0043005~neuron projection	14	6	4.6E-04
GOTERM_BP_FAT	GO:0031175~neuron projection development	12	4,8	8.0E-04
GOTERM_CC_FAT	GO:0042995~cell projection	20	8	0,0015
GOTERM_BP_FAT	GO:0030030~cell projection organization	14	6	0,0017
GOTERM_CC_FAT	GO:0001726~ruffle	6	2,4	0,0017
GOTERM_CC_FAT	GO:0031252~cell leading edge	8	3,2	0,0020
GOTERM_CC_FAT	GO:0005856~cytoskeleton	31	12,4	0,0027
GOTERM_BP_FAT	GO:0001667~ameboidal cell migration	4	1,6	0,0139
GOTERM_BP_FAT	GO:0030029~actin filament-based process	9	4	0,0179
GOTERM_BP_FAT	GO:0048858~cell projection morphogenesis	9	4	0,0194
GOTERM_BP_FAT	GO:0031346~positive regulation of cell projection organization	4	1,6	0,0263
GOTERM_BP_FAT	GO:0048812~neuron projection morphogenesis	8	3,2	0,0272
GOTERM_BP_FAT	GO:0031344~regulation of cell projection organization	5	2	0,0337
SP_PIR_KEYWORDS	Cytoskeleton	15	6	0,0368
GOTERM_BP_FAT	GO:0001755~neural crest cell migration	3	1,2	0,0390
GOTERM_BP_FAT	GO:0030048~actin filament-based movement	3	1,2	0,0390

Table S3. miR-146b-5p predicted target genes involved in migration and motility.

Functional Annotation Chart (DAVID) analysis of the miR-146b-5p predicted target genes returned several significantly enriched GO terms related to cell motility and migration (cytoskeleton, cell migration, actin filament-based processes and cell projections). Those GO terms were manually curated to create a list of genes (n=50) predicted to be targeted by miR-146b-5p that could be responsible for the observed phenotype.

Official Gene Symbol	Description	Entrez Gene ID
**ABL2**	v-abl Abelson murine leukemia viral oncogene homolog 2 (	27
**AKAP9**	A kinase (PRKA) anchor protein (yotiao) 9	10142
**ARL8A**	ADP-ribosylation factor-like 8A	127829
**BAG1**	BCL2-associated athanogene	573
**CAMSAP1**	calmodulin regulated spectrin-associated protein 1	157922
**CASK**	calcium/calmodulin-dependent serine protein kinase	8573
**CCDC6**	coiled-coil domain containing 6	8030
**CDC42BPA**	CDC42 binding protein kinase alpha (DMPK-like)	8476
**DCAF12**	WD repeat domain 40A	25853
**DCTN5**	dynactin 5 (p25)	84516
**DLGAP2**	discs, large (Drosophila) homolog-associated protein 2	9228
**DYNLL2**	dynein, light chain, LC8-type 2	140735
**EDNRB**	endothelin receptor type B	1910
**ERBB4**	v-erb-a erythroblastic leukemia viral oncogene homolog 4 (avian)	2066
**FAM65B**	family with sequence similarity 65, member B	9750
**GAS7**	growth arrest-specific 7	8522
**GDNF**	glial cell derived neurotrophic factor	2668
**GRIN2A**	glutamate receptor, ionotropic, N-methyl D-aspartate 2A	2903
**KIAA0284**	KIAA0284	283638
**KIF24**	kinesin family member 24	347240
**KIT**	Proto-oncogene tyrosine-protein kinase Kit	3815
**KLF7**	Kruppel-like factor 7 (ubiquitous)	8609
**LRP2**	low density lipoprotein-related protein 2	4036
**MAPT**	microtubule-associated protein tau	4137
**MYO5A**	myosin VA (heavy chain 12, myoxin)	4644
**MYO6**	myosin VI	4646
**NF2**	neurofibromin 2 (merlin)	4771
**NFASC**	neurofascin homolog (chicken)	23114
**NOS1**	nitric oxide synthase 1 (neuronal)	4842
**NRAS**	neuroblastoma RAS viral (v-ras) oncogene homolog	4893
**NRP2**	neuropilin 2	8828

**NUMB**	numb homolog (Drosophila)	8650
**ONECUT2**	one cut homeobox 2	9480
**PLEKHH2**	pleckstrin homology domain containing, family H member 2	130271
**PPM1A**	protein phosphatase 1A, magnesium-dependent, alpha isoform	5494
**PRC1**	protein regulator of cytokinesis 1	9055
**RABGAP1**	RAB GTPase activating protein 1	23637
**ROBO1**	roundabout, axon guidance receptor, homolog 1 (Drosophila)	6091
**SEPT3**	septin 3	55964
**SLC38A1**	solute carrier family 38, member 1	81539
**SLITRK3**	SLIT and NTRK-like family, member 3	22865
**SLITRK4**	SLIT and NTRK-like family, member 4	139065
**STRBP**	spermatid perinuclear RNA binding protein	55342
**STRN**	striatin, calmodulin binding protein	6801
**STX3**	syntaxin 3	6809
**SYT1**	synaptotagmin I	6857
**TLN2**	talin 2	83660
**TTL**	tubulin tyrosine ligase	150465
**WASF2**	WAS protein family, member 2	10163
**WASF3**	WAS protein family, member 3	10810

Table S4. List of the primers used in quantitative-PCR

Gene	Forward primer	Reverse primer
TAL1	AACAATCGAGTGAAGAGGAG	CTTTGGTGTGGGGACCAT
18S	GGAGAGGGAGCCTGAGAAACG	CGCGGCTGCTGGCACCAGACTT
pri-miR-146b	CTGGGAACGGGAGACGATTC	AAGTTGGGAGCCCAAACCAT

Table S5. Semi-quantitative 5-point scoring system for grading extent of leukemia cell infiltration in secondary organs.

NUMERICAL SCORE	DESCRIPTION	DEFINITION
**0**	absent	without leukemia cells in representative tissue sections
**1**	minimal	less than 1 tumor cell per high power field
**2**	mild	tumor cell infiltration is easily identified but of limited extent
**3**	moderate	infiltration is prominent but there is significant potential for increased severity
**4**	severe	infiltration occupies the majority of the organ
**5**	extreme	severe infiltration with extreme impact on neighboring organ/tissue architecture (exclusive for CNS infiltration)

Supplementary References

1. Griffiths-Jones, S., Saini, H.K., van Dongen, S. & Enright, A.J. miRBase: tools for microRNA genomics. *Nucleic Acids Res* **36**, D154-8 (2008).
2. Barata, J.T., Cardoso, A.A., Nadler, L.M. & Boussiotis, V.A. Interleukin-7 promotes survival and cell cycle progression of T-cell acute lymphoblastic leukemia cells by down-regulating the cyclin-dependent kinase inhibitor p27(kip1). *Blood* **98**, 1524-31 (2001).
3. Patnaik, S.K., Kannisto, E., Mallick, R. & Yendamuri, S. Overexpression of the lung cancer-prognostic miR-146b microRNAs has a minimal and negative effect on the malignant phenotype of A549 lung cancer cells. *PLoS One* **6**, e22379 (2011).
4. de Chaumont, F. *et al.* Icy: an open bioimage informatics platform for extended reproducible research. *Nat Meth* **9**, 690-696 (2012).
5. Lewis, B.P., Burge, C.B. & Bartel, D.P. Conserved seed pairing, often flanked by adenosines, indicates that thousands of human genes are microRNA targets. *Cell* **120**, 15-20 (2005).
6. Maragkakis, M. *et al.* Accurate microRNA target prediction correlates with protein repression levels. *BMC Bioinformatics* **10**, 295 (2009).
